# Supplementary material for: A galling insect activates plant reproductive programs during gall development
Source: Sci Rep. 2019 Feb 12;9:1833. doi: 10.1038/s41598-018-38475-6 (PMC6372598; doi:10.1038/s41598-018-38475-6)
Supplement: Supplementary file 1 — Table S1 [file 41598_2018_38475_MOESM1_ESM.pdf]

Title: A galling insect activates plant reproductive programs during gall development

Authors: Jack C. Schultz[1,2*], Patrick P. Edger[1,3], Mélanie J.A. Body[1,2], Heidi M. Appel[1,2]

Affiliations: (1) Division of Plant Sciences, Christopher S. Bond Life Sciences Center, University of Missouri, Columbia, MO 65211, USA

(2) Present address: Department of Environmental Sciences, Bowman-Oddy Laboratories, University of Toledo, Toledo, OH 43606, USA

(3) Present address: Department of Horticulture, Michigan State University and Ecology, Evolutionary Biology and Behavior, Michigan State University, East Lansing, MI 48824, USA

Legend: Table S1. Primer sequences and conditions used in ddPCR.

V. vinifera locus	*A. thaliana* ortholog	Gene ID	Gene name	Forward primer	Reverse primer	cDNA dilution	PCR volume (µL)	Annealing temperature (°C)	Number of cycles
GSVIVT01023102001	AT5G49720	DEC	DEFECTIVE CYTOKINESIS	5'-TGCGCCCTGAGGACATTGAT-3'	5'-CCTCGCTGCTCCCTAGAAAACTTA-3'	1/50x	10 µL	55 °C	40
GSVIVT01035724001	AT5G25530	DNAJ	Heat shock family protein	5'-CCGGTGGTAGTGTCAGGCTTAGG-3'	5'-CACCCGGGCTTCACTTCAATG-3'	1/100x	10 µL	55 °C	40
GSVIVT01021303001	AT4G18960	AG	AGAMOUS	5'-TCAAGCGGATCGAAAACACCACT-3'	5'-GCGCTTGCCTTCTTATACCTCTCA-3'	1/100x	10 µL	55 °C	40
GSVIVT01008655001	AT5G61850	LFY	LEAFY	5'-CAGAATGGGGTCGGCGGATGATAA-3'	5'-ACCGCGCTCTTTGGCAATGTTCTG-3'	1x	2 µL	55 °C	40
GSVIVT01027577001	AT2G45650	AGL6	AGAMOUS-LIKE 6	5'-AAGGAAGACCCAAATGATGATAGA-3'	5'-GTTCGGGTTCGCAGTCCA-3'	1/50x	5 µL	50 °C	40
GSVIVT01018951001	AT3G50870	MNP	MONOPOLE	5'-ACGATCGACGGGCTGGTTCTTGTG-3'	5'-GGCGGCGGCTGTGGCTCTC-3'	1/50x	5 µL	60 °C	40
GSVIVT01034073001	AT5G02030	PNY	PENNYWISE	5'-TTGCCTTCCGATTTGCTAACTTG-3'	5'-AGGGCTCCGGCTTGAACACC-3'	1/50x	5 µL	54 °C	40
GSVIVT01027579001	AT2G45660	SOC1	SUPPRESSOR OF OVEREXPRESSION OF CO 1	5'-CAACCGTATCAAGCTCCGAATCAA-3'	5'-CTCTCCCCTCAGGCAGTCCAATG-3'	1/100x	10 µL	54 °C	40
